# Contribution of Post-translational Phosphorylation to Sarcomere-Linked Cardiomyopathy Phenotypes

**DOI:** 10.3389/fphys.2016.00407

**Published:** 2016-09-14

**Authors:** Margaret V. Westfall

**Affiliations:** Department of Cardiac Surgery, University of MichiganAnn Arbor, MI, USA

**Keywords:** myofilament, post-translational modification, signaling, cardiomyopathy, phosphorylation

## Abstract

Secondary shifts develop in post-translational phosphorylation of sarcomeric proteins in multiple animal models of inherited cardiomyopathy. These signaling alterations together with the primary mutation are predicted to contribute to the overall cardiac phenotype. As a result, identification and integration of post-translational myofilament signaling responses are identified as priorities for gaining insights into sarcomeric cardiomyopathies. However, significant questions remain about the nature and contribution of post-translational phosphorylation to structural remodeling and cardiac dysfunction in animal models and human patients. This perspective essay discusses specific goals for filling critical gaps about post-translational signaling in response to these inherited mutations, especially within sarcomeric proteins. The discussion focuses primarily on pre-clinical analysis of animal models and defines challenges and future directions in this field.

## Introduction

More than 3800 gene mutations are linked to inherited cardiomyopathies (ICs) and identification of underlying gene mutations continues to expand (https://www.ncbi.nlm.nih.gov/clinvar/). Animal models expressing individual mutations have provided insight into the human disease and a better understanding of myofilament force transduction mechanisms (Tardiff, 2005, 2011). In these models, the pathophysiological response is often linked to a specific disease progression such as hypertrophic, dilated, restrictive, left ventricular noncompaction, and/or arrhythmogenic right ventricular phenotypes (Fatkin et al., 2014). However, understanding how a specific mutation leads to the cardiac phenotype remains a persistent question (van der Velden et al., 2015), and the factors contributing to disease variability in patients are only partially understood. One area which may provide insight into these issues, and therefore deserves further consideration, is dynamic local myofilament signaling and its impact on downstream networks and/or global signaling within cardiac myocytes. This Perspective focuses on the possibility that IC-linked mutations alter local myofilament signaling and contribute to downstream remodeling and disease progression. Our current understanding of dynamic post-translational myofilament signaling also is briefly summarized to lay the foundation for future work aimed at investigating relationships between IC-linked mutations and myofilament modulation.

**Figure 1:**
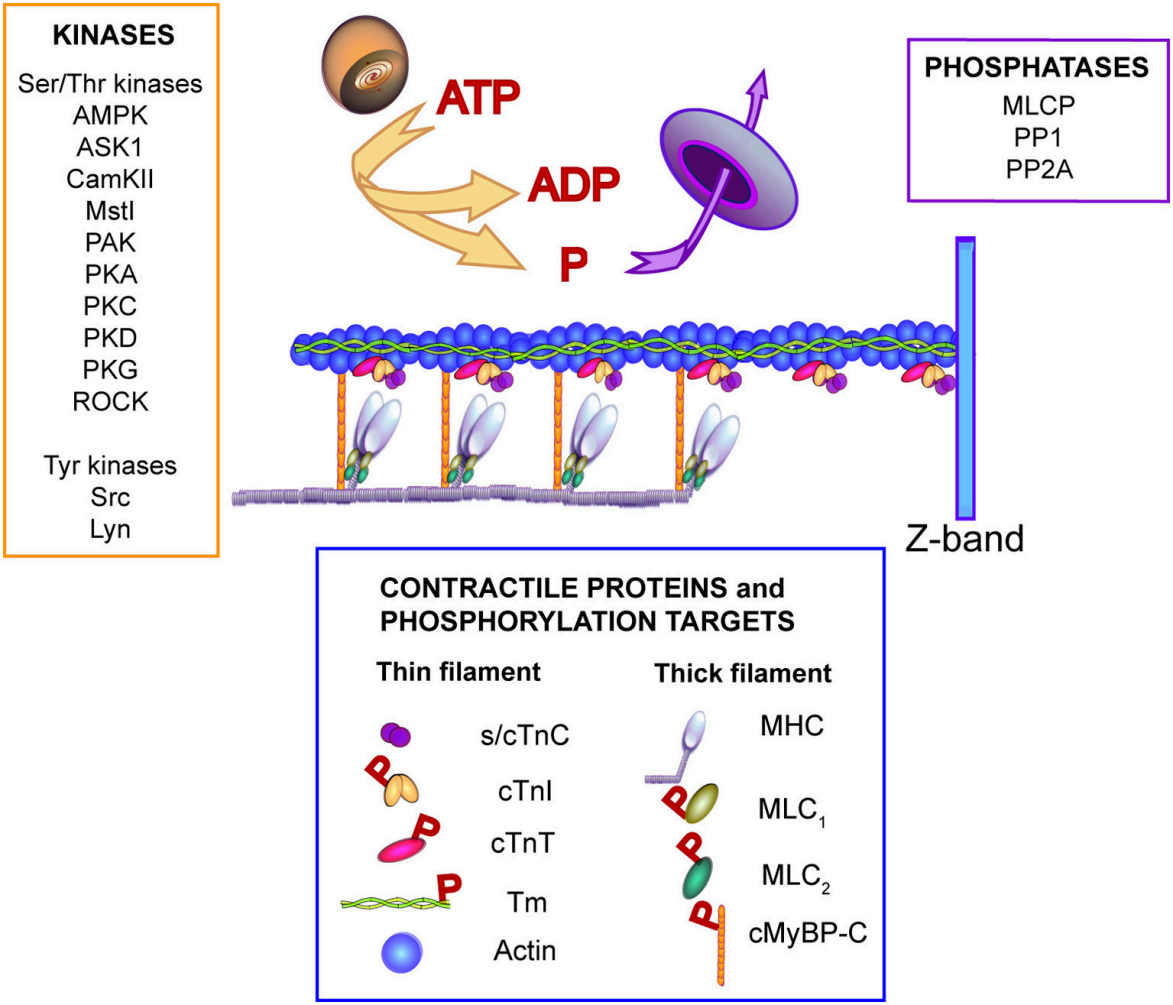
**Illustration of signaling kinases and phosphatases known to modulate phosphorylation of contractile proteins**. One or more of the kinases and/or phosphatases shown may contribute to secondary or “local” phosphorylation changes in response to IC-linked mutations. The Ser/Thr kinases shown to target myofilament proteins include adenosine monophosphate kinase (AMPK), apoptosis signal-regulating kinase 1 (ASK1), Ca^2+^/calmodulin protein kinase II (CamKII), sterile 20-like kinase 1 (Mst1), p21-activated kinase (PAK), protein kinase A (PKA), protein kinase C (PKC), protein kinase D (PKD), protein kinase G (PKG), and Rho-associated protein kinase (ROCK). Tyrosine kinases which target myofilament troponin I include non-receptor activated Src and the Lck/Yes novel (Lyn) kinase (Salhi et al., 2014). Phosphatases known to target myofilament proteins include myosin light chain phosphatase (MLCP), protein phosphatase I (PPI), protein phosphatase 2A (PP2A) (Solaro and Kobayashi, 2011). The contractile proteins shown in this illustration are slow/cardiac troponin C (s/cTnC), cardiac troponin I (cTnI), cardiac troponin T (cTnT), alpha-tropomyosin (Tm), and actin in the thin filament plus the myosin heavy chain (MHC), myosin light chains 1 and 2 (MLC_1_, MLC_2_, respectively), and cardiac myosin binding protein C (cMyBP-C). The proteins identified as phosphorylation targets include cTnI, cTnT, Tm, MLC_1_, MLC_2,_ and cMyBP-C (indicated by red P in the legend). For further information see the following references: (He et al., 2003; Barefield and Sadayappan, 2010; Solaro and Kobayashi, 2011; Streng et al., 2013; Westfall, 2014; Huang and Szczesna-Cordary, 2015).

First, it is important to point out that previous work in humans and animal models indicate IC-linked heart disease is complex. In patients, morbidity and mortality are often not easily explained by an identified mutation acting as a primary physiological insult or substrate (Ho et al., 2015). Instead, a temporal and spatial network of factors contributes to progressive cardiac structural and functional remodeling in IC patients, and can ultimately evolve into end stage heart failure. In addition to a primary mutation, factors known or suspected to increase the risk for disease include second hit and epigenetic mutations, polymorphisms, and other genetic modifiers, which include genes linked to cardiac remodeling, and environmental factors such as sex, aerobic activity levels, and risk factors such as hypertension (McNally et al., 2013; Månsson, 2014; Ho et al., 2015; van der Velden et al., 2015). In addition, cardiac remodeling and dysfunction is progressive on many levels and includes alterations in cellular morphology, signaling, and function, cell-cell architecture, plus organ-level electrical, and pump dysfunction. Signaling modulation is predicted to be an important focus for future work in recent reviews (Yar et al., 2014; van der Velden et al., 2015). Figure 1 illustrates the variety of signaling pathways known to phosphorylate myofilament proteins, and therefore, could contribute to modulation by targeting myofilament proteins for phosphorylation. The emphasis on myofilament modulation in this Perspective is based on the possibility that myofilament phosphorylation may be an early secondary response to IC-linked mutations, and therefore present prior to significant structural and functional remodeling within myocytes. Myofilament signaling may continue to contribute to adaptive functional responses and/or initiate one or more later compensatory behaviors associated with cardiac remodeling and disease, such as alterations in excitation-contraction (E-C) coupling, myocyte Ca^2+^ handling, and metabolism (Ashrafian et al., 2011).

In animal models expressing IC-linked mutations, E-C coupling and Ca^2+^ handling network alterations are often detected in parallel with *in vivo* evidence of cardiac performance compensation and/or dysfunction, and prior to end-stage heart failure (Ashrafian et al., 2011). These changes in Ca^2+^ increase the risk for developing arrhythmia and sudden cardiac death (Ashrafian et al., 2011; Yar et al., 2014), and the events responsible for initiating and/or causing remodeling of the Ca^2+^ signal may be critical for understanding IC-linked disease progression. Interventions to prevent or delay disease progression prior to the onset of Ca^2+^ remodeling would be desirable in high risk families and/or patients. However, little is known about the process or mechanism(s) responsible for the initiation of Ca^2+^ remodeling in these patients. IC-linked mutations may initiate changes in local myofilament signaling network(s) and the myofilament post-translational modification (PTM) pattern helps to maintain cardiac performance prior to any changes in Ca^2+^ handling. Evidence is accumulating that myofilament residues targeted by signaling pathways can initiate additional “secondary” or “adaptive” changes in the phosphorylation of other myofilament residues to modulate function (Montgomery et al., 2002; Scruggs et al., 2006; Lang et al., 2015), which appears to maintain steady state contractile function in the short term (Lang et al., 2015). Chronic activation of this secondary signaling within the myofilament may become inadequate and/or serve as a direct trigger for later structural and functional remodeling such as the IC-associated alterations in Ca^2+^ handling and E-C coupling described above. Although critical studies are still needed to prove this idea, future support for a direct role of local myofilament signaling in response to IC-linked mutations could lead to early diagnostic tests and/or therapeutic strategies to prevent or minimize IC disease progression in high risk patients.

## IC-linked mutations and a role for local myofilament signaling

There are some general observations which are consistent with a role for local myofilament signaling responses in IC-linked structural and functional remodeling. First, a causative mutation is usually not identified in new probands until cardiac dysfunction develops, which is often during adolescence or later (Cirino and Ho, 2008). The known impact of an IC-linked mutation on myofilament function and/or Ca^2+^ remodeling also may not predict the cardiac phenotype in animal models or patients, especially at early time points (Jacques et al., 2008; Jensen et al., 2013). A recent developmental study also demonstrated that inhibition of IC-linked gene expression during the first 6 weeks of life markedly reduced cardiac remodeling at 40 weeks in an α–MHC^R403Q^ mouse model, while more modest improvements developed if mutant protein expression was inhibited after 6 weeks of age (Cannon et al., 2015). Secondary modulatory mechanisms in the myofilament are consistent with these observations and could contribute to developmental lags and/or unexpected phenotypes. Myofilament modulatory networks also may undergo developmental transitions over the same perinatal period observed for many contractile proteins (Cummins, 1982; Lyons et al., 1990; Reiser et al., 1994, 2001; Suurmeijer et al., 2003). Impaired or altered myofilament signaling development could result in permanently sub-optimal myofilament modulation in adults with IC-linked mutations. Alternatively, this local signaling network modulation may be hard-wired to respond to myofilament perturbations such as IC-linked mutants, and either directly or indirectly trigger further adverse structural and functional remodeling of myocardium.

The local myofilament signaling concept also is supported by reported changes in the phosphorylation of multiple myofilament protein residues in response to IC-linked mutation expression, and alterations in additional phosphorylated residues linked to heart failure (Table 1). Altered myofilament phosphorylation develops in at least one IC-linked mutation for each contractile protein, and there is significant potential for myofilament phosphorylation to modulate contractile function based on the myofilament residues already identified as phosphorylation targets (see Table 1 and references). However, it is not known whether a given contractile protein or mutations clustered into a specific cardiomyopathy produce common spatial and/or temporal phosphorylation patterns. Thus, to test whether myofilament modulatory phosphorylation makes an early contribution to IC-linked phenotypes requires rigorous experimental testing in the future. As part of these studies, it is important to identify the dose-dependent spatial and temporal impact of each IC-linked mutation on myofilament phosphorylation and understand the modulatory impact of each phosphorylated contractile protein residue. Although not included here, phosphorylation of additional sarcomeric proteins, such as titin, also may contribute to this modulation. A few representative studies on cardiac troponin I (cTnI) mutations and phosphorylation are briefly presented below to illustrate our current understanding and the rationale for future directions on myofilament phosphorylation in response to IC-linked mutations.

**Table 1:** **Contractile protein phosphorylation sites associated with inherited cardiomyopathies (IC) and heart failure (HF)**.

Protein (Uniprot #)^*^	IC – linked sites^**^	Additional sites linked to HF^**^	**Additional putative sites**	**References**
Tropomyosin (P09493)	**S283**^1^			^1^(Warren et al., 2008; Marston et al., 2013; Schulz et al., 2013)
Troponin T (P45379-6)	**T203, T284**^2^	**T194, S198**^3^	S2, T275^3^	^2^Sfichi-Duke et al., 2010; Michael and Chandra, 2016 ^3^Reviewed by: Streng et al., 2013; Wei and Jin, 2011
Troponin I (P19429)	**S23/S24**^4^, S42, S44, T143^5^	**S5, S6, Y26, S42, S44, S77, T78, S166, T181, S199**^6^	T51, S150^6,7^	^4^Reviewed by: Messer and Marston, 2014 ^5^Burkart et al., 2003b; Kobayashi et al., 2004 ^6^Zhang et al., 2012 ^7^Nixon et al., 2012; Oliveira et al., 2012
cMyBP-C (Q14896)	**S275, S284, S304**^8^	**S286, T290**^9^	S18, S78, Y79, S86, S133, T274, S297, S311, S424, S427, T602, T607, S708, S106710	^8^van Dijk et al., 2012 ^9^Kooij et al., 2013 ^10^Jia et al., 2010; Kooij et al., 2013 Reviewed by: Barefield and Sadayappan, 2010
MLC1/2 (P08590/P10916)	**MLC_2_- S15**^11^	**MLC_1_- S195**^11^	MLC2-S1912	^11^Reviewed by: Huang and Szczesna-Cordary, 2015 ^12^Sanbe et al., 1999

* *Uniprot number for human protein; numbering includes Met1 residue*.

** *Bold font indicates direct evidence; Regular font indicates indirect evidence; Changes in IC-linked sites are also detected during HF*.

## IC-linked mutations and β-AR signaling in myofilaments

Previous work on β-adrenergic receptor (β-AR) signaling provides direct support for a role of local myofilament signaling in IC-linked changes in cardiac function. Several IC-linked mutations directly influence myofilament phosphorylation and/or β-AR signal transduction, as illustrated by representative cTnI mutations. Protein kinase A (PKA)-induced cTnI-S23/24 phosphorylation significantly contributes to the positive β-AR-induced lusitropic response (Takimoto et al., 2004; Yasuda et al., 2007; note that residue numbering is based on Uniprot human protein accession numbers, see Table 1). Uncoupling between the β-AR receptor and this response often develops in IC-linked animal models (reviewed by Messer and Marston, 2014). Poor outcomes are associated with myofilament β-AR uncoupling in other types of human heart failure, and the ability of IC-linked mutations to cause this uncoupling is proposed to be a prognostic indicator in patients with IC-linked mutations (Messer and Marston, 2014).

Several mechanisms can produce β-AR uncoupling in response to IC-linked mutations. Some IC-linked mutations directly disrupt post-translational cTnI-S23/24 phosphorylation. For example, the IC-linked cTnI-R21C mutation directly blocks PKA-induced phosphorylation of the adjacent S23/24 residues (Gomes et al., 2005; Wang et al., 2012; Dweck et al., 2014; Cheng et al., 2015). IC-linked mutations in more distant proteins also modify this PKA-targeted cTnI phosphorylation (Najafi et al., 2016). Alternatively, PKA continues to phosphorylate myofilament targets, such as cTnI-S23/24, in the presence of other IC-linked mutations. Representative mutations such as cTnI-R145G and -P82S, disrupt signal transduction within cTnI to cause β-AR uncoupling (Deng et al., 2001; Messer and Marston, 2014; Ramirez-Correa et al., 2015; Cheng et al., 2016). The cTnI-P82S mutation is noteworthy because the diastolic dysfunction and late-onset of disease in humans associated with this mutation is postulated to be a long-term consequence of secondary alterations in PKA-related myofilament signaling (Nimura et al., 2002; Mogensen et al., 2004; Frazier et al., 2008; Ramirez-Correa et al., 2015). In addition, IC-linked mutations may indirectly cause β-AR uncoupling due to changes in the overall myofilament phosphorylation status (Kooij et al., 2010), which could result from differences in other myofilament associated kinase and phosphatase activities (Figure 1).

## IC-linked mutations and additional myofilament signaling pathways

While β-AR induced PKA modulation is among the most studied signaling pathways targeting myofilaments, a number of additional signaling pathways also modulate myofilament function (Figure 1). A few studies also indicate that IC-linked mutations modify both kinase and phosphatase signaling pathways and downstream target residues other than β-AR/PKA-targeted sites. Mutation-related alterations associated with the protein kinase C (PKC) second messenger serve as a representative example. First, progressive increases in cardiac PKC expression and increased PKC affinity for sarcomeric proteins are associated with IC-linked mutations (Arimura et al., 2004; Sfichi-Duke et al., 2010). Modification of end-targets, such as PKC phosphorylation of cTnI-S42/44 provides some initial support. Myofilament function is similarly modified by either PKC-induced phosphorylation or phospho-mimetic cTnI-S42/44 substitutions (Noland et al., 1996; Burkart et al., 2003b). Interestingly, the myofilament response to phospho-mimetic cTnI-S42/44 is significantly greater in myofilaments expressing IC-linked tropomyosin (Tm)-E180G compared to controls (Burkart et al., 2003a).

Multiple neurohormones activate receptor-induced PKC signaling in myocytes, such as angiotensin II (AgII), endothelin, and catecholamine activation of α-adrenergic receptors (Dorn and Force, 2005). Accelerated and/or exaggerated cardiac remodeling and dysfunction develop in response to one or more of these neurohormones in mice with IC-linked mutations (Maass et al., 2004; Gramlich et al., 2009). This severe response has been interpreted as a stress response, but myofilament-associated PKC activity may already be modified in myofilaments expressing IC-linked mutations independent from receptor activation. Thus, further neurohormone stimulation of PKC may accelerate additional remodeling and produce progressive deterioration in cardiac function. This interpretation is supported by evidence of more severe remodeling in endothelin-treated, cardiac-derived stem cells from patients with IC-linked mutations (Tanaka et al., 2014). Pro-left ventricular polymorphisms present in the renin-angiotensin-aldosterone (RAA) axis also are associated with higher morbidity and mortality in patients with IC-linked mutations (Ortlepp et al., 2002; Kaufman et al., 2007). In addition, environmental stressors known to activate the RAA axis, such as pressure overload, further exacerbate IC-associated contractile dysfunction (Chen et al., 2013). While angiotensin receptor inhibitors failed to reverse fibrosis (Axelsson et al., 2015), some tangential evidence in Duchenne's muscular dystrophy (DMD) patients indicates earlier treatment with these types of inhibitors may be beneficial in treating DMD patients with cardiomyopathy (Duboc et al., 2007; Kamdar and Garry, 2016). DMD is caused by mutations in dystrophin, a crucial component of the costamere, which anchors sarcomeres to the sarcolemma.

Many of the potential signaling networks associated with myofilaments have the ability to produce a range of outputs via multi-layer signaling cascades capable of targeting both kinases and phosphatases, multiple sarcomeric protein targets, and multiple residues targeted within a single myofilament protein (Figure 1). The complexity of local myofilament signaling contributes to difficulties in defining the modulatory role for a given signal in myofilament function (Angeli et al., 2004). As a result, these signaling networks may not be easily recognized as contributors to IC-linked remodeling and/or disease. However, these types of pathways also are noteworthy because they are either predicted or known to act as oscillators capable of flexible outputs. Oscillatory signals have the potential to provide highly dynamic modulation to maintain steady state structure/function (Angeli et al., 2004). An IC-linked mutation could disrupt or alter one or more signals in an oscillatory pathway to produce subtle shifts in phosphorylation turnover at multiple target residues. These types of pathways may have little initial impact, but lead to bi- or multi-phasic temporal alterations in one or more target PTMs (Angeli et al., 2004). An IC-linked mutation which chronically induces secondary myofilament signaling to modulate function may either become inadequate to maintain myofilament structure and function, or directly trigger further adaptations beyond the myofilament, such as the myocyte Ca^2+^ handling modifications described earlier.

While a secondary reduction in myofilament phosphorylation can coincide with cardiac dysfunction (Bayliss et al., 2013; Alves et al., 2014), no published reports prove bifurcative/ oscillatory signaling and/or altered PTM levels develop in multiple myofilament proteins prior to detectable morphological and/or functional remodeling in animal models with IC-linked mutations. Short-term expression of cardiac troponin T (cTnT)-R92Q in bigenic mice provides some indirect evidence. These mice develop early alterations in a range of signaling pathways associated with structural remodeling, which returned to baseline after turning off mutant expression (Lutucuta et al., 2004). There is also some indirect support for IC-induced oscillatory changes based on changes in Ca^2+^ wave frequencies in cardiomyocytes expressing gain-of-function SHP-2/PTPN11 mutations, which are linked to Noonan's syndrome (Uhlén et al., 2006). In addition, myofilament PTMs and functional responses observed in adult myocytes after PKC gene transfer are consistent with bifurcative myofilament signaling (Hwang et al., 2013). These data alone do not provide adequate proof that local myofilament modulation contributes to IC-linked remodeling, but suggest that local myofilament signaling in IC-linked mice is worthy of analysis. Specifically, studies are needed to determine whether local myofilament modulation precedes and/or works in parallel with other adaptive responses associated with structural and functional remodeling observed in IC animal models.

## Dynamic signaling modulation in cardiac myofilaments

IC-linked mutations also may trigger a secondary signaling response, which may arise from structural changes imposed by a mutation. This secondary response could involve one or more signaling pathways known to target myofilament proteins (Figure 1). However, in contrast to the typical receptor-based signaling activation discussed earlier, only signaling networks localized to the myofilament undergo changes in activity. This localized signaling also may undergo dynamic changes in response to structural alterations produced during contraction and relaxation. Signaling studies utilizing phospho-mimetic and -null substitutions at myofilament target residues provide some initial support for the presence and role of dynamic, local myofilament signaling modulation (Lang et al., 2013, 2015). Specifically, alterations in the phosphorylation of other myofilament residues develops in myocytes expressing phospho-mimetic or non-phosphorylatable substitutions at one or more kinase-targeted myofilament residues (Montgomery et al., 2002; Scruggs et al., 2006, 2009; Lang et al., 2013; Nixon et al., 2014; Lang et al., 2015). This secondary phosphorylation also is associated with altered functional responses (Lang et al., 2013, 2015). Secondary adaptations in myofilament phosphorylation are reported in both thick and thin filaments using a variety of approaches. For example, a cTnI-S150 phospho-mimetic blunts the β-AR/PKA myofilament response (Nixon et al., 2014) and elevated myofilament phosphorylation develops in mice expressing non-phosphorylatable ventricular myosin light chain (MLC; Scruggs et al., 2009). Other post-translational modifications also trigger local myofilament phospho-modulation, as indicated by alterations in cardiac myosin binding protein C (cMyBP-C) phosphorylation after S-glutathiolation increases during heart failure (Stathopoulou et al., 2016). Taken together, these results are consistent with local myofilament modulatory signaling changes during sustained structural or functional perturbations in the sarcomere.

Other approaches, such as proteomic analysis of myofilament proteins during heart failure, also hint at dynamic PTM modulation within myofilaments. Heart failure is associated with altered phosphorylation residues in several contractile proteins (see Table 1; Dubois et al., 2011; Zhang et al., 2012; Kooij et al., 2013; Walker et al., 2013). Secondary signaling also is reported in some, but not all myofilament phospho-mimetic and -null animal models. These local signaling changes may contribute to phenotypic differences among these models, such as the significant differences reported in cTnI models with phosho-substitutions at PKC-targeted sites (Pi et al., 2002; Sakthivel et al., 2005; Bilchick et al., 2007; Kirk et al., 2009). While differences in genetic approach, mouse strain, age, and mutant expressivity may factor into these differences, highly organized signaling network(s) which locally modulate myofilament structure and function also may contribute to divergent phenotypes.

## Future directions

Future work needs to establish the circuitry, physiological functions, and temporal response of local myofilament modulatory signaling, and test whether this local modulation is an early or longer-term contributor to IC-linked remodeling and/or dysfunction. A parallel, translational goal for this work is the development of diagnostic tools, improved clinical management, and therapies to prevent and/or delay disease progression in IC patients. The integration of computational modeling, myofilament, cellular, and *in vivo* genetic model work is critical for achieving these goals. As a result, significant advancements are likely to depend on an unusually high level of cooperativity and resource sharing among investigators.

## Author contributions

The author confirms being the sole contributor of this work and approved it for publication.

### Conflict of interest statement

The author declares that the research was conducted in the absence of any commercial or financial relationships that could be construed as a potential conflict of interest.
